# Materia Medica Conference

**Published:** 1896-03

**Authors:** W. A. Dewey

**Affiliations:** Secretary, 170 W. 54th St., New York


### MATERIA MEDICA CONFERENCE.

Detroit, June 16th and 17th, 1896.

At the last meeting of the American Institute of Homœopathy a committee of three was appointed, "*To select a large committee of those interested in the Materia Medica, including several of our homœopathic specialists, to provide for the consideration and discussion of questions pertaining to the construction of a Scientific Materia Medica, and to call and arrange for a Materia Medica Conference in connection with the next session of this Institute, the Conference to continue one or more days (as may be found necessary), and to adjourn finally before the opening of the Institute session. The committee to report its papers and discussions to the Institute for its action.*"

This committee consisted of Drs. Pemberton Dudley, J. M. McClelland, and J. S. Mitchell.

The larger committee appointed by these gentlemen is composed of the following: Drs. T. F. Allen, E. H. Porter, M. Deschere, H. C. Houghton, and W. A. Dewey, of New York; Conrad Wesselhœft, of Boston; A. W. Woodward and H. C. Allen, of Chicago; Pemberton Dudley and B. F. Betts, of Philadelphia; Eldridge C. Price, of Baltimore; Millie J. Chapman, of Pittsburg; Harold Wilson, of Detroit; M. W. Vandenburg, of Fort Edward, and A. L. Monroe, of Louisville.

This committee held its first meeting on November 21st. A list of subjects was selected for the work of the first conference only, as the recommendation to appoint this committee included also a recommendation, "*That similar conferences should be held under the auspices of the Institute, from year to year, until we arrive at definite plans and methods for placing the Materia Medica upon a strictly scientific basis.*" Dr. T. F. Allen was chosen Chairman, and Dr. W. A. Dewey Secretary of the committee.

The committee desires to present the following programme: The Conference will meet at the place of the Institute meeting in Detroit, on Tuesday, June 16th, at 3 P. M., and hold three sessions. The first from 3 P. M. to 6 P. M., the second from 8 P. M. to 11 P. M., and the third on Wednesday, June 17th, from 10 A. M. to 1 P. M.

At these three sessions there will be presented and discussed the following topics:

I. Has the Law of Similars ever been unequivocally demonstrated by the deductions from general practice, and do we not require its more formal proof by inductive experimental research?

*Essayist,* Conrad Wesselhœft, M. D., Boston, Mass. *Discussions by* C. W. Butler, M. D., Montclair, N. J.; Martin Deschere, M. D., New York; Charles S. Mack, M. D., Chicago, and Charles Mohr, M. D., Philadelphia.

II. In what particulars has the proving of drugs deviated from the rules laid down by Hahnemann in *The Organon,* and in what particulars do Hahnemann's rules and directions for proving drugs differ from, or fall short of, those required by the methods and precautions of modern scientific research?

*Introductory Remarks,* T. F. Allen, M. D., New York. *Essayist,* Eldridge C. Price, M. D., Baltimore. *Discussions by* M. W. Vandenburg, Fort Edward, N. Y.; E. H. Porter, M. D., New York; Conrad Wesselhœft, M. D., Boston, and George Royal, M. D., Des Moines, Ia.

III. In the search for the simillimum, shall we indorse Section 8 of *The Organon,* which says that the totality of the symptoms must be the sole indication to direct us in the choice of a remedy?

*Essayist,* William Bœricke, M. D., San Francisco. *Discussions by* H. C. Allen, M. D., Chicago; W. J. Hawkes, M. D.,

Chicago; I. D. Buck, M. D., Cincinnati, Ohio, and L. C. McElwee, M. D., St. Louis, Mo.

The time limit for the above essays and the discussions thereon has been fixed as follows: Essays not to exceed thirty minutes; discussions must be limited to fifteen minutes. The essayist is to have an additional fifteen minutes in which he may comment on the matter presented in the discussions.

The balance of the time of each session may be occupied in general discussions of five minutes' duration each. As a large number undoubtedly will desire to discuss these important topics, and as the time will be limited, those who desire to take part in the discussions are invited to send their names to the Secretary; signifying the topics they wish to discuss. The remaining time of the sessions will then be allotted in the order in which such requests are made. Respectfully submitted:

Committee on Materia Medica Conference.

W. A. Dewey, M. D., *Secretary*, 170 W. 54th St., New York.

